# A New Node Deployment and Location Dispatch Algorithm for Underwater Sensor Networks

**DOI:** 10.3390/s16010082

**Published:** 2016-01-09

**Authors:** Peng Jiang, Jun Liu, Binfeng Ruan, Lurong Jiang, Feng Wu

**Affiliations:** 1 Key Lab for IOT and Information Fusion Technology of Zhejiang, Hangzhou 310018, China; liujunhdu@163.com (J.L.); ruanbinfeng@126.com (B.R.); fengwu@hdu.edu.cn(F.W.); 2 College of Automation, Hangzhou Dianzi University, Hangzhou 310018, China; 3 School of Information Science and Technology, Zhejiang Sci-Tech University, Hangzhou 310018, China; jianglurong@zstu.edu.cn

**Keywords:** underwater sensor networks, node deployment, greedy iterative strategy, location dispatch

## Abstract

Considering that deployment strategies for underwater sensor networks should contribute to fully connecting the networks, a Guaranteed Full Connectivity Node Deployment (GFCND) algorithm is proposed in this study. The GFCND algorithm attempts to deploy the coverage nodes according to the greedy iterative strategy, after which the connectivity nodes are used to improve network connectivity and fully connect the whole network. Furthermore, a Location Dispatch Based on Command Nodes (LDBCN) algorithm is proposed, which accomplishes the location adjustment of the common nodes with the help of the SINK node and the command nodes. The command nodes then dispatch the common nodes. Simulation results show that the GFCND algorithm achieves a comparatively large coverage percentage and a fully connected network; furthermore, the LDBCN algorithm helps the common nodes preserve more total energy when they reach their destination locations.

## 1. Introduction 

Recently, studies on wireless sensor networks have begun to focus on underwater sensor networks (UWSNs) [1,2]. These studies have mainly concentrated on solving the problems of node deployment, network routing, and node localization to accomplish the scheduled monitoring task, with full consideration of the special underwater environment [3]. Node deployment refers to the optimization of the network topology by adjusting the location of nodes in the network, and forming the desired network to meet the demands of monitoring at a relatively low deployment cost. The node deployment for UWSNs must fully consider the characteristics of the application environment. On the one hand, the monitoring targets are often the slowly-changing physics and chemistry indexes, such as the water pressure, water temperature, and water salinity. Thus, the demand for some of network performance factors, such as the network coverage rate, can be reduced. And sparsely deploying the nodes is feasible to reduce the deployment cost [4]. On the other hand, despite the spare distribution, the deployed nodes should be connected with the sink node in a single-hop or multi-hop manner to guarantee that the acquired sensing information can be delivered to the sink node, that is, the network must achieve full network connectivity. The quality of deployment determines whether the configuration of the limited resource, such as network energy or communication bandwidth, is reasonable. Deployment quality also affects the service quality of the network sensing and communication [5]. Therefore, designing the proper deployment strategy according to the application environment is necessary [6].

At the initial stage of the node deployment, the sensor nodes are usually scattered randomly on the surface of the water by plane or by ship. Random deployment refers to the random depths of the scattered nodes. Although this kind of deployment method is fit for underwater monitoring, it cannot guarantee excellent monitoring performance. If the nodes move to the destinations determined by some optimization algorithms, this kind of deployment method is called deterministic deployment, which has attracted much attention because of its better monitoring performance compared with random deployment [7,8]. Two main problems must be solved in deterministic deployment: (1) node deployment that refers to the process of designing the deployment strategy to calculate the destination set that consists of the prospective node destination locations, thus ensuring that the network comprising these nodes can obtain the desired network coverage rate and ensure full network connectivity; (2) node location dispatch that refers to the process of choosing the proper destination location from the destination set for each node that is randomly scattered on the water surface at the initial stage of node deployment, thus helping the nodes preserve more energy and prolonging network lifetime. The current study will focus on these two problems.

Regarding the node deployment problem, Alam *et al.* [9] have considered the coverage and connectivity issues of 3D networks, where the goal is to find a node placement strategy with 100% sensing coverage of a 3D space, while minimizing the number of nodes required for surveillance. The authors have proposed a regular node deployment based on the Voronoi tessellation of 3D space, and they have claimed that the Truncated Octahedron Deployment (TOD) algorithm is the best way. Although this algorithm can achieve full coverage and connectivity with dense distribution of nodes and the given relationship between the node sensing range and communication range, it is not appropriate for the sparse distribution application characteristic of UWSNs. Dhillon *et al.* [10] proposed the Maximum Average Coverage Deployment (MACD) algorithm by using the grid model to describe the monitored space, and executed node deployment through greedy iterative strategy. Although this algorithm can be applied to UWSNs, if node density is small, it cannot achieve full network connectivity and can only achieve a low network coverage rate. Pompili *et al.* [11] have proposed deployment strategies for two-dimensional and three-dimensional communication architectures for UWSNs, and the objective is to determine the minimum number of sensor nodes to be deployed to achieve optimal sensing and communication coverage. However, the strategies call for large number of nodes, which is not proper for the application where the distribution of nodes is sparse. Halder *et al.* [12] pointed out that, for WSNs comprised of imaging sensor nodes or underwater sensor nodes, since the network sensing performance is significantly affected by their positions or the sensor nodes are relatively expensive, the deterministic deployment is as a better way to deploy the network than the random way. Moreover, based on the regular hexagonal cell, the authors proposed a non-uniform, location-wise pre-determined node deployment strategy, which can balance the energy consumption and prolong the network lifetime.

In this study we propose a Guaranteed Full Connectivity Node Deployment (GFCND) algorithm. In the GFCND algorithm, the nodes are logically divided into two types: coverage nodes and connectivity nodes. First, the GFCND algorithm attempts to deploy the coverage nodes according to the greedy iterative strategy, after which the connectivity nodes are used to improve the connectivity of the network to make the whole network fully connected. Compared with the TOD and MACD algorithms, the GFCND algorithm can achieve a comparatively large coverage percentage and a fully connected network.

In the node location dispatch problem, the node location dispatch of UWSNs is similar to that of Hybrid Wireless Sensor Networks (HWSNs) [13]. To decrease the movement energy consumption during the dispatch of the latter, Wang *et al.* [14] proposed a type of heuristic algorithm, which divides the dispatch process into a few rounds, each including two phases: the Pareto optimization phase and the spanning tree construction phase. However, this type of dispatch algorithm is based on the premise that all moving nodes must be connected with the sink node, which is not feasible in solving the node location dispatch problem in UWSNs. Furthermore, Wang *et al.* [15] proposed the Central Dispatch (CD) algorithm and the Distributed Dispatch (DD) algorithm. Compared with the random dispatch algorithm, these two algorithms can decrease the movement energy consumption during the location dispatch. However, the CD algorithm needs a fully connected network at the beginning of the dispatch process, which cannot be realizable in UWSNs, because the sensor nodes are usually scattered randomly on the surface of the water by the plane or ship, possibly producing isolated nodes. In addition, the DD algorithm demands that all the nodes must communicate with the neighboring nodes at a certain interval, which produces a large number of communications.

To resolve the node location dispatch problem, a Location Dispatch Based on Command Nodes (LDBCN) algorithm is proposed in this study. This algorithm accomplishes the location adjustment of the common nodes with the help of the SINK node and the command nodes. Then, the command nodes dispatch the common nodes. This algorithm helps the common nodes preserve more total energy when they reach their destination locations. 

The contributions of this study can be summarized as follows:

(1) To resolve the node deployment problem, we propose a novel deployment algorithm that can achieve a comparatively large coverage percentage and a fully connected network with relatively sparse node distribution.
(2) To resolve the node location dispatch problem, we propose a novel node location dispatch algorithm based on the command nodes, which can help the common nodes preserve more total energy when they reach their destination locations.

The rest of this paper is organized as follows: we describe the algorithms in detail in Section 2, then we provide the simulation evaluation in Section 3, and finally, we present our conclusions in Section 4.

## 2. Principle of the Algorithm

### 2.1. The Overall Layout

#### 2.1.1. Assumptions and Directions

Inspired by the similar assumptions in [16], which solves the node non-uniform deployment problem for UWSNs, all the nodes can freely move in all directions and can locate themselves. Their moving speed is the same and denoted as *vn*. Moreover, the coordinates information of the monitored water space has been stored in the memories of nodes before the initial random scattering. The nodes can be classified into three types. The first type is the sink node, which is singular, collects and analyzes the information from other nodes, and completes the overall location adjustment of other nodes. In the GFCND algorithm, the sink node is at the center of the water surface. In the LDBCN algorithm, the sink node is randomly scattered on the water surface and then moves to the center of the water surface. The second type of node is the command node, of which four are used. The communication range of this node is the same as that of the sink node. The command node is only involved in the LDBCN algorithm, and these nodes solve the problem of the full connectivity at the initial stage of the node deployment and to finish the location dispatch of the common nodes. The command nodes are first randomly distributed on the water surface and then move to the predetermined locations to connect with the sink node. The third type of node is the common node denoted as *comn*; its function is to finish the conventional monitoring task. All common nodes have the same sensing range and communication range, and they can be logically divided into two types: the coverage node and connectivity node. The coverage node is denoted as *cv*; its major function is to improve the network coverage rate, whereas its minor function is to improve the network connectivity rate. Meanwhile, the connectivity node is denoted as *cn*; its major function is to improve the network connectivity rate, whereas its minor function is to improve the network coverage rate.

#### 2.1.2. Algorithm Layout

As described in the Introduction section, the study of the node deterministic deployment problem for UWSNs includes two aspects: the problems of node deployment and node location dispatch. First, in the node deployment problem, we propose the GFCND algorithm and logically divide the common nodes into coverage nodes and connectivity nodes. Then, we calculate the locations of the coverage nodes in a greedy iterative strategy. Afterwards, the connectivity nodes are used to improve network connectivity to make the whole network fully connected. Thus, we can obtain the destination set *des*, which consists of the prospective node destination locations. Then, *des* is stored into the memory of all the nodes (including the sink node, the command nodes, and the common nodes). After all the nodes are scattered randomly on the water surface, choosing a proper destination from the destination set *des* for each node is required to prolong the network lifetime. This process helps the nodes preserve more energy after they move along a straight line from the initial random locations on the water surface to their corresponding destinations. Considering that this problem is also defined as a node location dispatch problem, we design the corresponding node location dispatch algorithm, *i.e.*, the LDBCN algorithm. The algorithm accomplishes the location adjustment of the common nodes with the help of the SINK node and the command nodes. Then, the command nodes dispatch the common nodes, and choose the proper destination location from the destination set *des* for the common nodes to help them preserve the total energy as much as possible after they reach their destinations along a straight line.

### 2.2. Models and Definitions

#### 2.2.1. Models

##### (1) 3D Underwater Space Model

**Figure 1:**
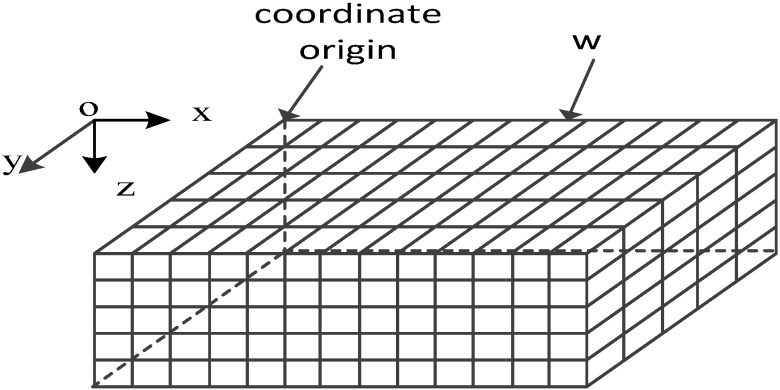
Coordinate system for UWSNs.

The 3D underwater space is a large cube divided into a number of small cubes whose side length is *w* (also known as the cube resolution, Figure 1). All the small cubes have selected center points to represent themselves. The coordinates of cube *p_j_* are (*a_j_,b_j_,c_j_*).

##### (2) Node Probabilistic Sensing Model

The probabilistic sensing model proposed in [17] by Wan *et al.* is adopted in the current study to describe the coverage of node *s_i_* on cube *p_j_*, which can be denoted as the following formula:

(1) $$c_{p}(s_{i},p_{j})=\left\{ \begin{array}{l} 0,\;if\;r+r_{e}\leq d(s_{i},p_{j})\;\\ e^{-\lambda \alpha^{\beta }},\;if\;r-r_{e}<d(s_{i},p_{j})<r+r_{e}\;\\ 1,\;if\;r-r_{e}\geq d(s_{i},p_{j})\; \end{array}\right.$$

where α = *d*(*s_i_, p_j_*) denotes the Euclidean distance between node *s_i_* and cube *p_j_*; λ and β are the coefficients related with the sensing probability, whose values are set to be 0.5 and 1, respectively; *r* is the sensing range; and *r_e_* is the error of sensing range. For cube *p_j_*, the joint sensing probability describing the possibility of being sensed by a few nodes can be calculated with the following formula:

(2) $$c_{p}(p_{j})=1-\prod_{s_{i}\in V_{p}}\left(1-c_{p}(s_{i},p_{j})\right)$$

where *V_p_* denotes the set that consists of the node *s_i_*, whose distance away from cube *p_j_* is not larger than *r* + *r_e_* . If the joint sensing probability of cube *p_j_* is not less than the value of *R_p_* (*i.e.*, the network coverage rate threshold), cube *p_j_* is considered to be covered. The value of *R_p_* depends on the application environment; if the application has low requirements for the network coverage rate, *R_p_* can be set a value less than 1 [18].

##### (3) Dispatch Model Based on Dispatch Nodes

**Figure 2:**
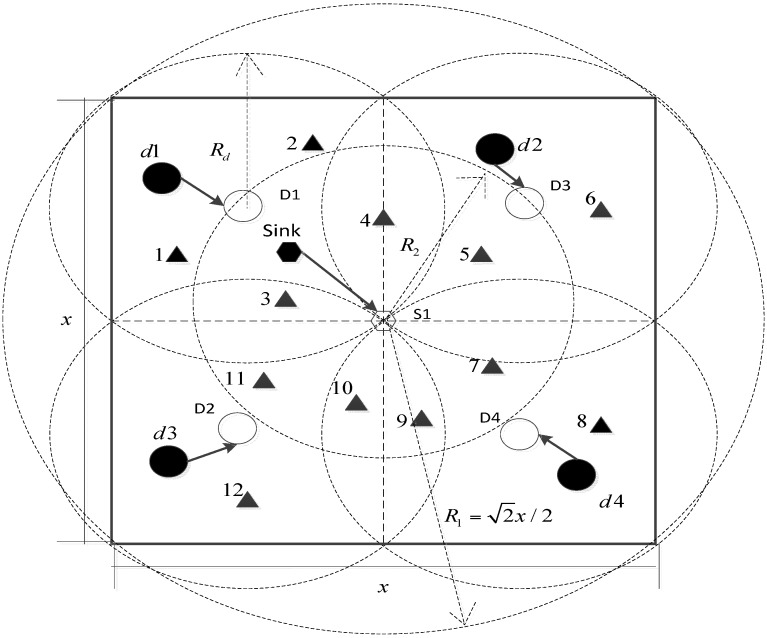
Dispatch model based on dispatch nodes.

Figure 2 shows the dispatch model based on dispatch nodes from the overlook view. Though the common nodes should be located in the 3D monitored underwater space, the main node location dispatch process happens on the water surface, which is a 2D scenario. 

As can be seen, the water surface is a square area whose side length is *x*; the dotted line means that the square area is divided into four smaller squares, in which each command node is responsible for administering the corresponding small square. At the initial stage of the dispatch process, all nodes are scattered randomly on the water surface. The solid hexagon denotes the sink node, and the hollow hexagon represents the destination location of the sink node. The solid circle denotes the command node, and the hollow circle represents the destination location of the command node. The solid triangle denotes the common node. Supposing that both the communication ranges of the sink node (*i.e.*, *R_2_*) and the command node (*i.e.*, *R_d_*) are $\sqrt{2}\times x/4$, and the sink node and the command nodes reach their own destinations, each common node is connected with at least one of the command nodes and each command node is connected with the sink node. Therefore, the problem of full network connectivity for the CD algorithm can be overcome as the network can now achieve full network connectivity at the initial stage of the dispatch process. When the scale of the monitored water space becomes large, the sink node and dispatch nodes can increase their communication ranges correspondingly to achieve full communication coverage on the water space surface. If the scale has increased to be so large that although the sink node and dispatch nodes reach their maximum communication ranges, they cannot achieve full communication coverage, the dispatch model and corresponding algorithm cannot be applied any more. However, compared with the existing CD algorithm, the dispatch model and algorithm proposed in this paper can enlarge the maximum water surface area from ${\left(R_{2}/\sqrt{2}\right)}^{2}$ to ${\left(2\sqrt{2}\times R_{2}\right)}^{2}$, resulting in a 15-fold improvement and of large practical significance. 

#### 2.2.2. Definitions

##### (1) Neighboring Cube Point Set

For the cube point, its neighboring cube point set can be the set that consists of the cube points whose distance away from itself is not bigger than *r* + *r_e_*. The neighboring cube point set of cube point *p_j_* can be denoted as the following formula:

(3) $$N(p_{j})=\{p_{i}|\;d(p_{i},p_{j})\leq r+r_{e}\}$$

where *d(p_i_*, *p_j_)* denotes the Euclidean distance between cube *p_i_* and cube *p_j_*.

##### (2) Network Coverage Rate

The network coverage rate, *CoR*, denotes the ratio of the number of cube points whose joint sensing probability is not smaller than *R_p_* and the total number of cube points. The network coverage can be calculated as the following formula:

(4) $$CoR=\frac{\sum_{j=1}^{G}DP\left(p_{j}\right)}{G}$$

where *G* denotes the total number of cube points, and the *DP(p_j_)* can be calculated as the following formula:

(5) $$DP(p_{j})=\{\begin{array}{c} 1\;C_{p}(p_{j})\geq R_{p}\\ 0\;C_{p}(p_{j})<R_{p} \end{array}$$

##### (3) Network Connectivity Rate

The network connectivity rate, *CnR*, is defined as the ratio of *M* and *N*, where *M* is the number of common nodes that can communicate with the sink node through single-hop or multi-hop communication, and *N* is the total number of common nodes. *CnR* can be calculated using the following expression:

(6) $$CnR=\frac{M}{N}$$

if the network connectivity rate is 1, the network achieves full network connectivity, and all the common nodes can communicate with the sink node through single-hop or multi-hop communication.

### 2.3. Description of the Problem and the Algorithm

#### 2.3.1. GFCND

##### (1) Problem Description

When expensive underwater sensor nodes are used to monitor the slowly-changing physics and chemistry indexes in the water space, the distribution of nodes is usually sparse, and the deployment algorithm should achieve a relatively high network coverage rate and ensure full network connectivity. Although some scholars have proposed different kinds of node deployment algorithms, such as the TOD algorithm proposed in [9] and the MACD algorithm proposed in [10], they cannot meet the above requirements with sparse node distribution. To solve such a problem, this study proposes the GFCND algorithm. In the GFCND algorithm, the nodes are logically divided into two types: coverage nodes and connectivity nodes. First, GFCND attempts to deploy the coverage nodes through greedy iterative strategy, after which the connectivity nodes are used to improve the connectivity of the network to make the whole network fully connected. Thus, we can obtain the destination set *des*, which consists of the prospective node destination locations. Then, *des* is stored into the memory of all the nodes (including the sink node, the command nodes and the common nodes).

##### (2) Algorithm Description

The common nodes are logically divided into two types: coverage nodes and connectivity nodes. First, the coverage nodes are deployed until the number of deployment times is equal to the number of coverage nodes. Supposing that the number of cube points covered in the neighboring cube point set *N(P)* of cube point *P* is *U(P)*, then *U(P)* can be calculated using the following formula:

(7) $$\{\begin{array}{c} U(P)=\sum_{v\in N(P)}F(v)\\ F(v)=\{\begin{array}{c} 1\;c_{p}(v)\geq R_{p}\\ \;0\;c_{p}(v)<R_{p}\; \end{array} \end{array}$$

**Figure 3:**
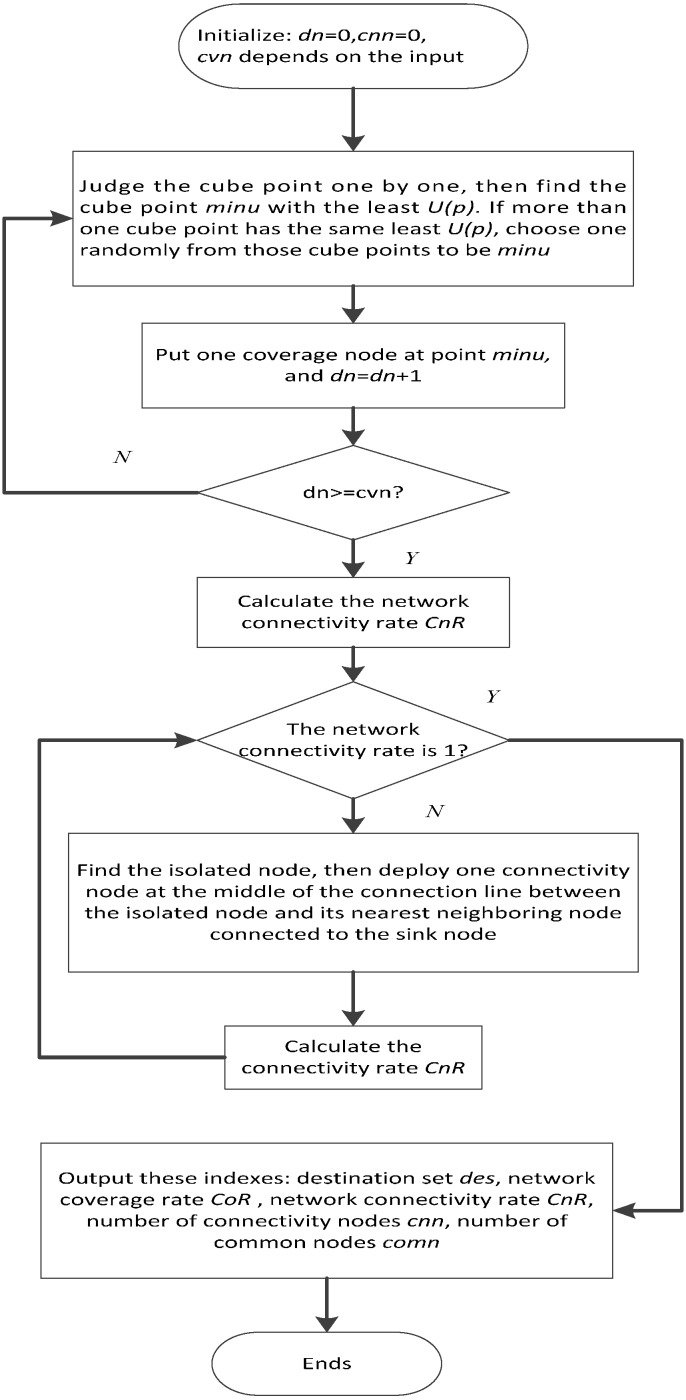
Flow chart of GFCND algorithm.

In each deployment, the coverage node should be deployed at the cube point *minu* whose *U(P)* value is the smallest. Because for the cube point *minu*, its *U(P)* value is the smallest, which means that it has the most neighboring cube points whose *F(v)* values are 0. In other words, it has the most cube points uncovered in its neighboring cube point set, and if a coverage node is placed at this cube point, the current deployment will gain maximum improvement. If more than one cube point has the same smallest *U(P)* value, then a random cube point among them is chosen as the place where the coverage node is located, indicating that the first coverage node is placed randomly. In each deployment, we aim to gain the maximum improvement in the current deployment, thus ignoring the overall optimization in the whole deployments. The determination of the cube point *minu* in the current deployment depends on the condition of the last deployment. This kind of deployment algorithm is called the greedy iterative strategy. After completing the deployment of the coverage nodes, the connectivity nodes are then deployed to improve the network connectivity rate according to the following method. First, the connectivity condition of each deployed coverage node is judged individually. Then, if any coverage node *S_1_* is disconnected with the sink node through the single-hop or multi-hop way, we find the nearest node *S_2_* connected to the sink node from the neighbor of node *S_1_*. Finally, we deploy a connectivity node at the middle of the connection line between the node *S_1_* and node *S_2_*. This deployment procedure of the connectivity node will continue until the network achieves the full connectivity. Afterwards, we can obtain the destination set *des*, which consists of the prospective node destination locations. Then, *des* is stored into the memory of all the nodes (including the sink node, the command nodes and the common nodes). The whole process of the GFCND algorithm is shown in Figure 3. In Figure 3, *dn* presents the number of the coverage node deployment times. *cnn* and *cvn* presents the number of the coverage nodes and connectivity nodes respectively, and *comnn* presents the number of the common nodes.

#### 2.3.2. LDBCN

##### (1) Problem Description

**Figure 4:**
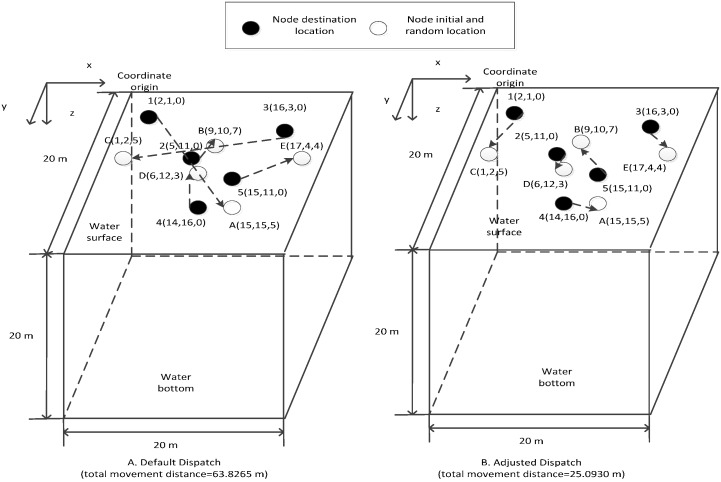
Total movement distance comparison for default and adjusted dispatches.

After calculating the destination set *des* with the GFCND algorithm mentioned in Section 2.3.1 and storing the set into the memory of all the nodes, the nodes can then be scattered randomly on the water surface by plane or ship. Given the randomness of the initial sowing, the common nodes may be far from their default destination locations. If each common node moves to its default destination location, the total movement distance of all the common nodes may be too large. For example, supposing that five nodes are scattered on the surface of the monitoring water space, which is a 20 m × 20 m × 20 m cube, the ID numbers and the initial locations of these nodes are 1(2,1,0), 2(5,11,0), 3(16,3,0), 4(14,16,0), 5(10,11,0) (the unit of measurement is m). Their respective default destination locations are *A*(15,15,5), *B*(9,10,7), *C*(1,2,5), D(6,12,3), and *E*(17,4,4) (the unit of measurement is also m). If these five nodes are required to move their default destination locations, that is, 1→A, 2→B, 3→D, and 5→E, the total movement distance is 63.8265 m (as is shown in Figure 4A). If these nodes comply with the following dispatch rules, that is, 1→C, 2→D, 3→E, 4→A and 5→B, the total movement distance is only 25.0930 m (as is shown in Figure 4B), which is a dramatic decease. Therefore, designing the proper node location dispatch strategy to choose the proper destination location from the destination set for each common node affects their total movement distance during movement from their initial random locations to their destination locations. This design further affects the total remaining energy of the common nodes when they reach their destination locations. To solve this problem, Wang *et al.*, proposed the CD and DD algorithms. However, the CD algorithm needs a fully connected network at the beginning of the dispatch process, whereas the DD algorithm demands that all the nodes must communicate with the neighboring nodes at a certain interval, which produces an overabundance of communications. Hence, the current study proposes the LDBCN algorithm, which accomplishes the location adjustment of the common nodes with the help of the sink node and command nodes. Then, the command nodes determine the optimized destination locations for the common nodes in its administrating area by utilizing the KM algorithm [19]. After receiving the calculation result from their corresponding command node, each common node moves to the destination location from their initial random location along the straight line.

#### 2.3.3. Algorithm Description

After calculating the destination set *des* with the GFCND algorithm mentioned in Section 2.3.1 and then storing the set into the memory of all the nodes, the nodes can be scattered randomly on the water surface by plane or ship. The LDBCN algorithm is run to choose the proper destination location from the destination set for each common node. This process is elaborated below.

*Step 1*: The sink node and the command nodes move to their destination locations, as is shown in Figure 2.

*Step 2*: Each common node sends the register information *ri* to its nearest command node. If the number of its nearest command nodes is more than 1 (meaning more than 1 command nodes owns the same shortest distance to this common node), the common node chooses the command node with smaller ID number. Then, each command node can know the number of the common nodes (*i.e.*, *ndn*) and the number of the destination locations (*i.e.*, *den*) in its administrating area. Hence, the command node calculates the match information *mi* and sends *mi* to the sink node:

(8) mi(i)=ndn(i)−den(i)       i=1,2,3,4

*Step 3*: After receiving the match information *mi*, the sink node demands the command node, whose *mi* is bigger than 0, to transport the common node to the area administrated by the command node whose *mi* is smaller than 0. The detailed process can be seen in Algorithm1.

*Step 4:* Each command nodes chooses the proper destination location for each of the common nodes in its administrating area. After receiving the calculation result from their corresponding command node, each common node moves to the destination location from their initial random location along the straight line. The detailed process can be seen in Algorithm 2.

**Algorithm 1.** Adjustment process of sink node.(I)The command nodes with the maximum and minimum *mi* are denoted as *madn* and *midn,* respectively. If more than one command node owns the same maximum or minimum *mi*, the one with the smaller ID number is denoted as *madn* or *midn*.(II)The sink node demands that the command node *madn* transports the common nodes in its administrating area to the administrating area of the command node *midn*. To decrease the movement distance, the common nodes nearest the command node *midn* are chosen for transport. Every time one common node is transported, the *mi* of the command node *madn* decreases by 1, and the *mi* of the command node *midn* increases by 1. This process continues until the *mi* of the command node *madn* or *midn* becomes 0. The transport process is as follows: the dispatch node *madn* chooses the common node nearest to the command node *midn*, and marks it as node *trn*. Meanwhile, *madn* sends the message *di* to node *trn*. The message *trn* contains the information about the command node *midn*. As soon as this message is received, node *trn* moves toward the command node *midn* along the connection line form itself to the command node *midn*, and examines its own location. If it reaches the administrating area of the command node *midn*, it stops moving.(II)The sink node judges whether the *mi* of all the command nodes is equal to 0 or not. If it is, the adjustment ends. Otherwise, (I) is repeated.

 

**Algorithm 2.** Dispatch process of dispatch nodes.After Step 3, the number of the common nodes is equal to the number of the destination locations in the administrating area of each command node. Therefore, each command node can dispatch the common nodes to the destination locations in its administrating area in a centralized manner. Taking any of the command nodes as an example, we suppose that the destination location set in its administrating area can be denoted as *L* = {*l*_1_,*l*_2_,…,*l*_k_}, and the set of common nodes is denoted as *S* = {*s*_1_,*s*_2_,…,*s*_k_}, and *el_i_* is the remaining energy of the *i*th common node. Then, the dispatch problem shifts to choosing the proper destination locations for these *k* common nodes on the condition $\max\sum_{s_{i}\in S,l_{j}\in L}(el_{i}-e_{m}\times d(s_{i},l_{j}))$, where e*_m_* is the energy cost per movement distance, and *d*(*s_i_,l_j_)* is the distance between the common node *s_i_* and the destination location *l_j_*. If we construct a bipartite graph *G* = (*S* ∪ *L*, *S* × *L*) based on the set *S* and *L*, and assume that the edge whose endpoints are *s_i_* ∈ *S* and *l_j_* ∈ *L* is denoted as *E*(*s_i_,l_j_)* and weight is defined to be *w*(*s_i_,l_j_)* = *el_i_–e_m_* × *d*(*s_i_,l_j_)*, the dispatch problem can be converted to an optimal match problem. The KM algorithm described in [19] is utilized to solve the abovementioned problem. After the calculating result is broadcast to the common nodes, the common nodes move to the destination location from their initial random location along the straight line.

## 3. Simulation Evaluation

### 3.1. GFCND

#### 3.1.1. Simulation Scenario

The TOD algorithm [9] is one of the existing typical regular node deployment algorithms, so it is chosen as the comparison to help verifying the performance of the GFCND algorithm. Since the MACD algorithm [10] is one of the existing typical deployment algorithms using a greedy iterative strategy, it is chosen as one of the comparison algorithms. MATLAB software is used to simulate the algorithms. The monitoring water space is a large cube with a volume of 90 m × 90 m × 54 m. The sink node is located at the center of the water surface, whose coordinates are (45, 45, 0) (the unit of measurement is m). The cube resolution *w* is 6 m, and the network coverage threshold *R_p_* is 0.6. The sensing range *r*, error of sensing range *r_e_*, communication range *Rt* are 12 m, 4 m and 16 m. respectively. Supposing that the numbers of coverage nodes (*i.e.*, *cvn*) are 10, 15, 20, 25, 30, 35, 40, 45, 50, 55, 60, 65, and 70 in the simulation, we first study the relationship between the numbers of the coverage nodes and those of the connectivity nodes. Then, we compare and evaluate the performances of the MACD, TOD and GFCND algorithms from the perspectives of network coverage rate and network connectivity rate.

#### 3.1.2. Simulation Results and Analyses

**Figure 5:**
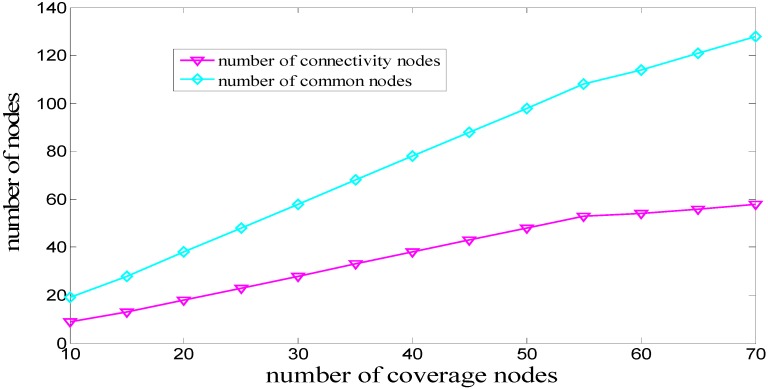
Comparison of the number of various nodes in the GFCND algorithm.

The simulation is conducted as the process shown in Figure 3. The numbers of coverage nodes (*i.e.*, *cvn*) are 10, 15, 20, 25, 30, 35, 40, 45, 50, 55, 60, 65, and 70. To achieve full network connectivity, the number of the connectivity nodes that must be deployed are 9, 13, 18, 23, 28, 33, 38, 43, 48, 53, 54, 56 and 58, respectively. The common nodes consist of the coverage nodes and the connectivity nodes. Thus, the numbers of common nodes are 19, 28, 38, 48, 58, 68, 78, 88, 98, 108, 114, 121 and 128, respectively, as shown in Figure 5. With the increase in the number of coverage nodes, the number of the connectivity nodes also has to increase to achieve full network connectivity. When the number of the coverage nodes is larger than 55, the increasing tendency of the connectivity nodes becomes increasingly smaller. The reason is that when the number of the coverage nodes is initially small, and then increases subsequently, more and more nodes become isolated and more connectivity nodes are needed to improve network connectivity. When the number of the coverage nodes comes to a relatively high value, the connectivity among nodes becomes stronger and the increase in the number of connectivity nodes becomes increasingly smaller.

**Figure 6:**
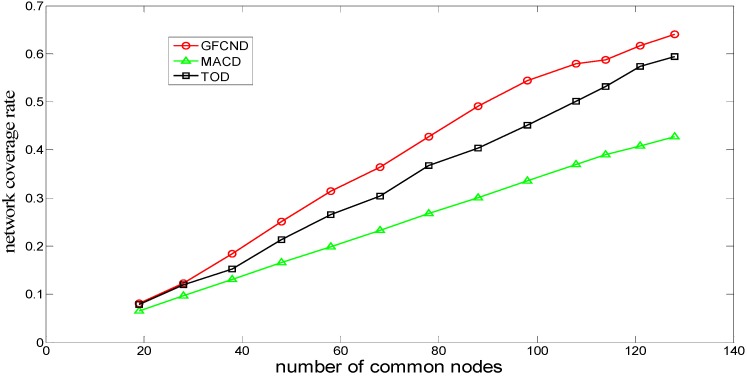
Network coverage rate for deployment algorithms.

Figure 6 shows the comparison of the relationship between the network coverage rates and the number of common nodes for the MACD, TOD, and GFCND algorithms. If the number of the common nodes is the same, the network coverage rate of the GFCND algorithm is always bigger than those of the MACD and TOD algorithms. The reason is that, in the GFCND algorithm, each of the coverage nodes is located at the cube point *minu* with the least *U(P)*, thus resulting in large network coverage rate improvement. The TOD algorithm can utilize the truncated octahedron Voronoi tessellation method to deploy the nodes in a regular way, as well as a deterministic way, and the MACD algorithm can utilize the greedy iterative strategy to deploy the nodes in a deterministic way. However, both of them try to improve the coverage rate from the overall perspective, ignoring improving the coverage contribution of each individual node, so though they can improve the network coverage rate to some extent, their advantages cannot be totally revealed when the number of the common nodes is small.

**Figure 7:**
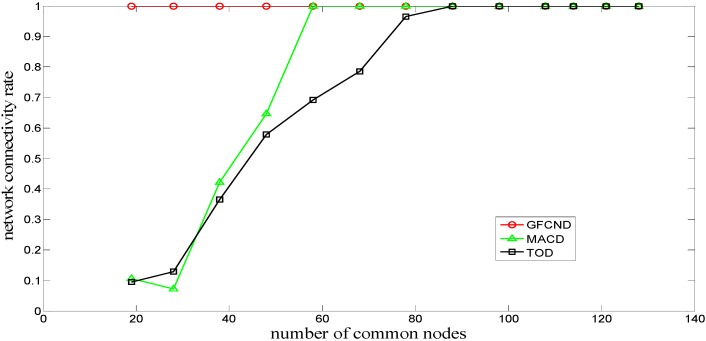
Network connectivity rate for deployment algorithms.

Figure 7 shows the comparison of relationship between the network connectivity rates and the number of common nodes for the MACD, TOD, and GFCND algorithms. As can be seen, the GFCND algorithm can consistently achieve full network connectivity. The reason is that the GFCND algorithm divides the common nodes into coverage nodes and connectivity nodes. The coverage nodes are then used to improve the network coverage rate, and the connectivity nodes are used to improve the network connectivity rate until the network achieves full connectivity. And it is also shown that when the number of the common nodes reaches a certain level, the MACD and TOD algorithms can also achieve full network connectivity. The reason is that both of them belong to the deterministic deployment algorithms and can optimize the locations of the common nodes to some extent.

### 3.2. LDBCN

#### 3.2.1. Simulation Scenario

MATLAB is used to simulate the algorithms. The monitoring water space is a large cube with a volume of 90 m × 90 m × 54 m. The communication range (*i.e.*, *R_t_*) of nodes is 16 m, and other related parameters are shown in Table 1. The destination set *des* is calculated using the GFCND algorithm mentioned in Section 3.1, and it can be stored into the memory of all nodes. Afterwards, all the nodes can be scattered randomly on the water surface, and the LDBCN algorithm is utilized to complete the dispatch of the common nodes. We then compare and evaluate the performances of the LDBCN, CD, and DD [15] algorithms from the perspectives of total communication times, average node movement distance, and average remaining energy of common nodes. To eliminate the effects of simulation randomness, we conduct the simulation 30 times and calculate the average as the final result, and choose 0.95 as the confidence level.

**Table 1:** Parameter settings of three dispatch algorithms.

Initial energy Initial energy of Initial energy of Node movement of sink node (J) command node (J) common node (J) speed (m/s)
CD 70000 / 50000 0.1
DD / / 50000 0.1
LDBCN 70000 40000 50000 0.1
Energy consumption Energy consumption Communication per movement distance (J/m) per communication (J) interval (s)
CD 50 20 /
DD 50 20 20
LDBCN 50 20 /

#### 3.2.2. Simulation Results and Analyses

Table 2 shows the comparison of the total communication times of common nodes during the entire dispatch process for the LDBCN, CD, and DD algorithms. As shown in Table 2, if the number of common nodes is the same, the total communication times for the DD algorithm is much bigger than those of the two other algorithms. The reason is that the DD algorithm demands that the common nodes should communicate with their neighboring nodes at a certain interval during the dispatch process, thus increasing the total communication times dramatically.

**Table 2:** Total communication times of common nodes for three dispatch algorithms.

Number of Common Nodes	CD	Confidence Intervals of CD	DD	Confidence Intervals of DD	LDBCN	Confidence Intervals of LDBCN
19	57	(42, 72)	4375	(4075, 4675)	91	(68, 114)
28	84	(69, 99)	7426	(7133, 7719)	122	(100, 144)
38	114	(100, 128)	13703	(13418, 13988)	169	(148, 190)
48	144	(130, 158)	21028	(20747, 21309)	213	(192, 234)
58	174	(161, 177)	24761	(24485, 25037)	246	(227, 265)
68	204	(192, 216)	36912	(36642, 37182)	286	(268, 304)
78	234	(222, 246)	45476	(45214, 45738)	334	(317, 351)
88	264	(252, 276)	49817	(49567, 50067)	372	(356, 388)

**Figure 8:**
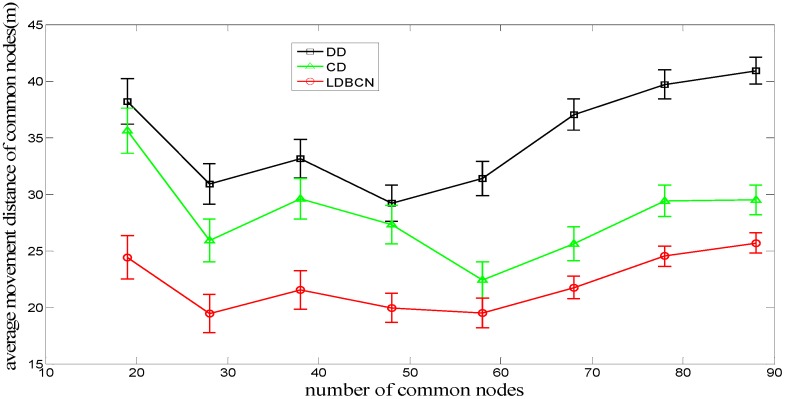
Average movement distance of common nodes for dispatch algorithms.

Figure 8 shows the comparison of the relationship between the average node movement distance of common nodes during the entire dispatch process and the number of common nodes for the LDBCN, CD, and DD algorithms. When the numbers of common nodes are equal, the average node movement distance of common nodes for the DD algorithm is the largest, followed by that of the CD algorithm and that of the LDBCN algorithm, which is the smallest. The reason is that in the DD algorithm, the sink node does not participate in the dispatch process, and the common nodes cannot obtain the global information of nodes and destinations. As a result, some nodes move toward the same destination during the dispatch process, making these nodes compete with each other. The loser has to move toward another destination, increasing the movement distance. In the CD and LDBCN algorithms, the competition between the common nodes can be eliminated and the node movement distance can be decreased, because the sink node or the command node can broadcast the global information and dispatch the destination locations that do not conflict with each other for the common nodes. Compared with the CD algorithm, the LDBCN algorithm divides the monitoring space into four smaller monitoring spaces with the help of the four command nodes; thus, the node movement scope and the node movement distance can be decreased.

**Figure 9:**
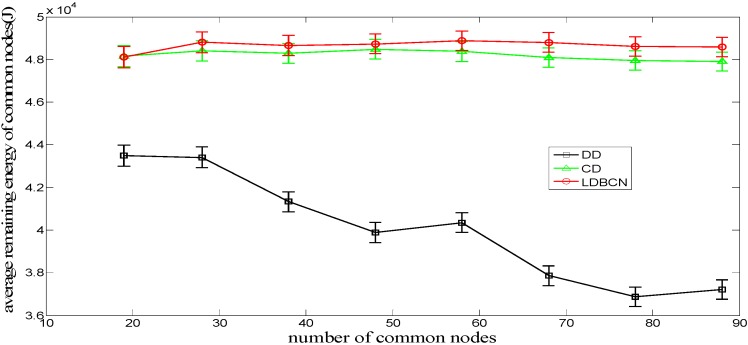
Average remaining energy of common nodes for dispatch algorithms.

Figure 9 shows the comparison of the relationship between the average node remaining energy of common nodes during the entire dispatch process and the number of common nodes for the LDBCN, CD, and DD algorithms. When the numbers of common nodes are equal, the average node remaining energy of common nodes for the LDBCN and CD algorithms are larger than that for the DD algorithm. The reason is twofold. First, the DD algorithm demands that the common nodes communicate with their neighboring nodes at a certain interval during the dispatch process, which may increase the communication energy consumption. Second, because the common nodes lack the global instruction during the dispatch process, the node movement distance may increase owing to possible competition for the same destination, thus increasing the movement energy consumption. Compared with the CD algorithm, the average communication times of the common nodes for the LDBCN algorithm has no large difference, and the average node movement distance is smaller. Therefore, for the LDBCN algorithm, the common nodes can complete their location dispatch with less energy consumption, allowing them to reserve more total energy after they reach their destinations.

## 4. Conclusions

Node deployment is one of the basic problems for UWSNs, and a proper deployment strategy should be designed according to the special underwater application environment characteristics, such as 3D network structure, limited node energy that is difficult to compensate, relative sparse node distribution, and so on. Compared with random deployment, deterministic deployment can achieve better network performance. This study focused on the problem of node deployment and node location dispatch in deterministic deployment. For the former problem, a novel deployment algorithm that can guarantee full network connectivity (*i.e.*, the GFCND algorithm) is proposed. In the GFCND algorithm, the nodes are logically divided into two types: coverage nodes and connectivity nodes. First, the GFCND algorithm attempts to deploy the coverage nodes according to the greedy iterative strategy, after which the connectivity nodes are used to improve network connectivity to make the whole network fully connected. Thus, the problem of obtaining the desired network coverage rate and ensuring full network connectivity is solved. After calculating the destination set *des* with the GFCND algorithm, for the following node dispatch problem, this study proposes a node dispatch algorithm based on the command nodes (*i.e.*, the LDBCN algorithm). The LDBCN algorithm accomplishes the location adjustment of the common nodes with the help of the SINK node and the command nodes. Then, the command nodes dispatch the common nodes, during which the KM algorithm is used to decrease the communication and movement energy consumption of common nodes, thus enabling them to preserve more total energy for other tasks after they reach their destinations. Considering the node drift or death in real underwater environments, how to solve the problem of network topology adjustment is one of our future research directions. Moreover, how to design the proper transmission model to describe the communicating through underwater acoustic signal also deserves our future research.
